# Synaptic input patterns triggering local dendritic spikes *in vivo*

**DOI:** 10.1186/1471-2202-16-S1-P95

**Published:** 2015-12-18

**Authors:** Lea Goetz, Martine R Groen, Arnd Roth, Michael Häusser

**Affiliations:** 1 Wolfson Institute for Biomedical Research, University College London, London, WC1E 6BT, UK

## 

Theoretical modelling and experiments *in vitro *have shown that the computations performed by single neurons critically depend on the spatiotemporal patterns of synaptic input to the neuron [1,2]. While recording the spike output of neuronal populations *in vivo *is now routinely possible, the spatiotemporal pattern of synaptic inputs to an entire neuron is still largely inaccessible to experiment. However, recent experiments in which sensory-evoked local dendritic spikes were observed *in vivo *have provided valuable constraints on possible spatiotemporal patterns of synaptic input [3-5]. For example, the synaptic input to layer 2/3 pyramidal neurons in mouse primary visual cortex evokes dendritic spikes at high frequencies during visual stimulation at the preferred orientation, but not at non-preferred orientations [3]. Here, we use a biophysical model to explore which spatiotemporal patterns of synaptic inputs can drive the observed spikes, and in particular the high frequencies in dendritic spike bursts.

First, we adapted a detailed active compartmental model of a neocortical layer 2/3 pyramidal neuron [3] to reproduce biophysical properties and firing statistics observed *in vivo *[6], such as firing threshold, mean membrane potentials for UP and DOWN states, and sparse action potential firing in the presence of Poisson distributed background synaptic input. The model generates fast dendritic spikes heterogeneous in amplitude, time course and spatial extent, as observed in *in vivo *experiments [3]. The dendritic spikes, together with somatic action potential firing, are abolished by blocking the NMDA receptor-mediated conductance, and their frequency is reduced by hyperpolarization via the dendritic recording site, again as observed in experiments [3].

Next we analyzed which spatiotemporal synaptic input patterns precede the generation of local dendritic events. We find that local spikes are preferentially triggered by excitatory synapses which are spatially and temporally clustered in the local dendritic branch and neighbouring branches. While there is great variability in the spatial distribution of synapses triggering local dendritic spikes, temporal patterns of excitatory synaptic input are stereotyped and tend to be sparse. High-frequency dendritic spikes similar to those observed *in vivo *occur preferentially when excitatory synaptic inputs are interspersed with inhibitory synaptic inputs. Furthermore, local spikes preceding backpropagating action potentials lead to the highest instantaneous dendritic spike frequencies. The highest sustained frequencies are generated by local spikes initiated sequentially in several neighbouring dendritic branches. Notably, only some of the high frequency dendritic events are effective in evoking action potential output, while others remain local. We use cluster analysis to establish the local and global conditions under which specific spatiotemporal patterns of synaptic input can influence neuronal output.

In summary, we have derived spatial and temporal rules for synaptic input and identify input patterns that locally exploit the non-linear integration capacities of dendrites. Which computations can be implemented by spiking dendrites receiving such synaptic input patterns? These constraints on synaptic input patterns, together with the realistic intrinsic properties displayed by the model, put us in a position to investigate the role of dendritic excitability in shaping the input-output relation of the neuron.
